# Expanding the use and interpretation of patient-centric cardiovascular clinical trial endpoints

**DOI:** 10.1017/cts.2025.10054

**Published:** 2025-07-03

**Authors:** Shelby D. Reed, Pishoy Gouda

**Affiliations:** 1 Duke Clinical Research Institute, Duke University, Durham, NC, USA; 2 University of Alberta, Edmonton, Alberta, Canada

**Keywords:** Patient-centered outcomes, patient-centric outcomes, weighted endpoints, clinical trials, patient preferences, net benefit

## Abstract

Significant improvements have been achieved to enhance the patient-centricity of clinical research, including the development and utilization of novel clinical trial endpoints. These include endpoints that harness outcomes that are important to patients and reflect the patients’ lived experiences. This may take the form of utilizing variables such as days alive and out of hospital (DAOH) and quality-of-life adjusted outcomes. The use of composite outcomes can be used to enrich patient-centricity by weighting or ranking events. These approaches have several nuances that should be considered including selecting appropriate events, defining outcomes, how to elicit or construct weights, and whose opinions to consider. After weights have been determined, a variety of approaches exist to combine weights with outcomes and make comparisons between groups. The approaches, including the win ratio, weighted win ratio, desirability of outcome ranking (DOOR), multicriteria decision analysis (MCDA), and variations of time-to-first composite event analyses, have unique advantages and challenges depending on the clinical scenario. While improving patient-centric outcomes is of high importance to multiple stakeholders, more comparative work is needed to characterize the implications of alternative approaches.

Stakeholders across academia, industry, regulatory and funding bodies, and patient advocacy have embraced increasing patient-centricity in clinical research. Significant strides are being realized in lowering the barriers to research participation through virtual study visits, reducing the need for in-person testing, increasing reliance on electronic health records and wearables, and broadening the use of social media platforms to enhance patient engagement. Those involved in planning and executing clinical trials can revel in making remarkable progress over a short period.

Despite this transformation, many trials rely on endpoints that are not patient-centric. Trials evaluating treatments for cardiovascular disease often rely on composite endpoints of a combination of episodic events, (e.g., all-cause or cardiovascular deaths, myocardial infarctions, strokes, revascularization procedures, and hospitalizations). Further complicating this aspect is that these combinations, the definitions of outcomes, and cutoff values vary across trials. Although each of the events may be indicative of a treatment’s impact, they are not equally important to patients, making it difficult to compare treatments within or across trials [1,2].

Analytic approaches introduce further problems. Hazard ratios, which are widely used to compare rates of composite endpoint events between treatment groups, typically only account for the timing of the first endpoint event for each patient regardless of its relative importance. For example, assume that two patients are randomized on the same day and that the outcome of interest is a composite of hospitalization for heart failure and death. A patient hospitalized for heart failure at 3 months is counted as worse off than a patient who dies anytime thereafter. If one patient dies and another is hospitalized for heart failure on the same day, they are treated equally in the analysis. Because the timing of events trumps the relative importance of different clinical outcomes in conventional analysis, the interpretation of study findings based on composite endpoints summarized using hazard ratios can be challenging.

The aim of this review is to provide an overview of the spectrum of patient-centric clinical trial outcomes and to broaden awareness about methods to elicit weights from stakeholders, analytical approaches, and ongoing challenges within this field. Throughout the review various concepts and terms are used and briefly defined, with more detailed explanations provided in Table 1.

**Table 1.** Common terms in patient-centric outcomes

Term	Explanation
Composite endpoint	Composite endpoints are a type of clinical trial outcome that combines multiple individual events. In atherosclerotic trials, these commonly include events such as cardiovascular death, myocardial infarction, and stroke. This approach is meant to increase the number of events that contribute to an estimated treatment effect to decrease the required sample size.
Weighted composite endpoint	One of the criticisms of composite endpoints is that different components do not have equal importance to neither patients nor physicians. Weighted composite endpoints aim to circumvent this by assigning relative weights to each component of the composite endpoint.
Patient-centric endpoints	Traditional clinical trial outcomes focus on severe clinical events, such as death, heart attack, or stroke. However, patients and physicians frequently disagree on the relative importance of disease outcomes. Patient-centric endpoints can take many forms and involve engaging with patients to determine relative importance of outcome events as well as varying levels of functional capacity or quality-of-life metrics.
Days alive and out of hospital	Days alive and out of hospital measures the number of days that an individual is both alive and out of hospital, which can be a useful metric in assessing chronic conditions that result in frequent hospitalizations. A limitation of this approach is that it counts hospital days as the same as days following a death.
Finkelstein-Schoenfeld test	A statistical method to compare two treatment groups when multiple event types occur. This involves ranking participants based on the severity of events experienced and subsequent pairwise comparisons to assess differences between treatment groups.
Win Ratio	An analytic approach that expresses the magnitude of treatment effect as an odds ratio, estimated as the number of “wins” divided by the number of “losses” when comparing a study intervention versus a control. With these methods, all patients in both study groups are initially compared to one another on the outcome that is chosen as the most important with three possibilities: a win, a loss, or a tie.
Global rank composite	An analytic approach where all participants are ranked based on prespecified hierarchy of outcomes, which can include a range of clinical outcomes, continuous biomarker values and quality-of-life metrics (e.g., death, hospitalization for myocardial infarction, troponin rise, symptoms of angina).
Ordinal composite endpoint	An endpoint for which multiple outcomes are summarized into a single measure that preserves the order of severity among outcomes.
Net benefit metric	Method of quantifying the comparative risks and benefits of a therapy versus a comparator by incorporating the rates of a beneficial versus harmful outcomes.
Desirability of outcome ranking	Analytic framework to evaluate clinical trial outcomes, by ranking them based on desirability from a given stakeholder's perspective.
Best-worst scaling	A method of eliciting patient preferences, where a small set of items are shown in a list, and participants are asked to identify the two most extreme items, in terms defined by the researcher (e.g., most and least important).
Generalized pairwise comparisons	An analytic approach that compares all pairs of patients across treatment groups. The win ratio is an example.
Discrete choice experiments	A survey-based method that quantifies patient preferences based on respondents’ selections among sets of hypothetical scenarios described using varying levels across a set of attributes (e.g., clinical outcomes, adverse events, treatment burden).
Multicriteria decision analysis	An approach designed to assist with decision making that evaluates many competing factors by assigning a weight to each component, combining them with estimates of relative performance on each component, and aggregating them into an overall score.
Monte Carlo simulation	Computational technique that uses repeated random sampling to model uncertainty and variability.

## Advances to improve patient-centricity

Clinical researchers have offered a variety of proposed approaches to improve patient-centricity of clinical trial results. One approach is to conduct secondary analyses, also using hazard ratios, to evaluate whether treatment effects are consistent across various outcomes. Another approach expands endpoint events beyond episodic medical events to include health-related quality-of-life measures. However, these findings are often overlooked if a treatment fails on the primary trial endpoint. Other advances have focused on capturing the “patient journey” through the use of endpoints like cumulative days alive and out of hospital (DAOH) [3,4]. In addition to providing a more holistic perspective about a treatment’s benefits, an additional advantage of a continuous, cumulative endpoint like DAOH is that treatment effects are reported as absolute differences that are preferable for communication with patients as opposed to relative differences in counts or rates [5]. Unfortunately, DAOH implicitly treats deaths and hospitalizations as equally important. Very few patients would be expected to agree. However, some limitations can be overcome by adjusting DAOH for quality of life or other metrics of functional capacity, which then takes into consideration not only DAOH but also symptom burden during those days [6]. This is especially important when we consider that individuals with lived experiences of chronic diseases, such as heart failure, value their quality of life on a day-to-day basis more than a hospitalization [7].

## Rank-based methods

A significant step forward in improving patient-centricity in clinical trials is the use of analytic approaches that explicitly incorporate the importance of events included in composite endpoints. Recent advances for handling multiple endpoints often incorporate a hierarchical ranking of events. The Finkelstein-Schoenfeld test [8] and win ratio [9] are increasingly being used in cardiovascular trials. The Finkelstein-Schoenfeld test generates a p-value for a null hypothesis, and the win ratio approach provides an additional magnitude of treatment effect interpreted as an odds ratio, estimated as the number of “wins” divided by the number of “losses” when comparing a study intervention versus a control. With these methods, all patients in both study groups are initially compared to one another on the outcome that is chosen as the most important with three possibilities: a win, a loss, or a tie. For example, using death as the most important outcome, for a given patient pair, if the patient in the intervention group dies and the patient in the control group is still alive on that date, the paired comparison is counted as a loss for the intervention group. If the patient in the intervention group is still alive and being followed when the patient in the control group dies, the paired comparison is counted as a win. If both patients in the pair die on the same date or live beyond a common follow-up period, the paired comparison is counted as a tie. All patient pairs counted as ties are then compared on the second most important outcome. The process continues until wins, losses, and ties have been determined for the remaining pairs for each outcome included in the composite endpoint. Finally, the total numbers of wins, losses, and ties are summed across outcomes, and the summary win ratio is reported along with a 95% confidence interval.

A global rank composite is similar to an unmatched win ratio [10], where all participants are ranked based on prespecified hierarchy of outcomes, which can include a range of clinical outcomes, continuous biomarker values and quality-of-life metrics (e.g., death, hospitalization for myocardial infarction, troponin rise, symptoms of angina). These ranks are subsequently analyzed using Wilcoxon–Mann–Whitney rank-sum test (U test). In an ordinal composite endpoint, multiple outcomes are summarized into a single measure, with participants assigned to the highest prespecified outcomes. For example, in a stroke trial participants may be categorized based on the occurrence on the worst observed (death = 1, major stroke = 2, minor stroke = 3, no stroke = 4). However, results from this analytic approach can be challenging to implement and interpret such as when introducing patient-reported outcomes.

Another alternative metric used to summarize wins and losses used in win ratios is the net benefit metric which is computed by subtracting the number of losses from the number of wins and dividing by the total number of patient pairs. Compared to the win ratio, the net benefit better conveys the magnitude of benefit because it accounts for the number of ties in the denominator, thereby resulting in a smaller measure of net benefit when there are many ties. Analysts are also applying nonparametric tests, like a Wilcoxon rank-sum test [11] and time-to-event analyses to compare ranked hierarchical endpoints.

Despite the advantages of generalized pairwise comparison approaches, they also suffer some shortcomings. Only when two patients tie on an endpoint does the hierarchical process continue and compare patients on subsequent, lower-ranked endpoints. Win ratios can also be influenced by the duration of follow-up. Because multiple events can occur across time, the treatment group with more wins can change over time (unless proportional hazards hold) [12]. Nevertheless, methods using hierarchical endpoints invite investigators to expand their scope to more patient-centric endpoints such as patient-reported measures and other continuous measures like 6-minute walk distance. However, in trials where there are few differences between treatment groups on higher-ranked endpoints, small differences between patients on lower-ranked, continuous endpoints could represent the majority of wins counted in a win ratio. One way to address this potential problem is to set a threshold value to define a win or a loss that could correspond to a minimally clinically important difference using a patient-reported outcome like the Kansas City Cardiomyopathy Questionnaire [13]. The EMPULSE trial evaluated the benefit of empagliflozin versus placebo in acute heart failure specified a hierarchical endpoint composed of all-cause death, number of heart-failure events, time-to-first heart-failure event, and ≥5-point change in the KCCQ total symptom score [14]. Even when using a threshold to address trivial differences in a continuous outcome, lower-ranked outcomes can drive between-treatment comparisons. Across both treatment arms in EMPLULSE, 6.2% died, 12.6% had a heart failure event, and 6.4% of the patient-to-patient comparisons were tied [14]. A contribution index analysis revealed that about two-thirds of the wins were attributable to differences in the KCCQ score despite secondary analyses revealing no significant difference in KCCQ-TS scores of ≥10 points at 90 days and an adjusted mean difference of 4.45 points, which is just short of the 5- to 10-point difference that is often cited for conveying a clinically important change [13,15].

Most clinical trials that have used generalized pairwise comparisons or ranking methods have relied on clinicians to rank order the endpoints despite studies reporting that patients and physicians vary in their views of the relative importance of endpoint events [16]. Interestingly, approximately half of composite endpoints used in major clinical trials exhibit a large gradient of perceived importance to patients among the individual components of the outcome [1]. Even if it is widely agreed that death is a more significant event than a hospitalization, trialists acknowledge that the rank order of other endpoints can be largely subjective and hard to establish.

An additional limitation of ranking approaches described above is that neither combinations nor quantities of lower-ranked events can compensate for the occurrence of a higher-ranked event. This limitation can be overcome only with more complicated rules for determining wins and losses. For instance, if two patients died on the same number of days following randomization and one patient had a stroke and the other did not, a rule that accounts for deaths and strokes could assign the patient without a stroke as a win. In coronary trials, because myocardial infarction is generally higher ranked than ischemia-driven revascularization, a patient who experienced a myocardial infarction at any point will be scored as a loss compared to a patient who underwent repeated revascularization procedures for angina.

Methods that account for the frequency of or the magnitude of gains for lower-ranked outcomes could be advantageous. For example, it may be preferable to allow several ischemia-driven revascularization procedures to outweigh a myocardial infarction or to allow large gains in health-related quality of life to offset a higher-ranked event such as a heart-failure hospitalization. The desirability of outcome ranking (DOOR) represents a meaningful step in this direction [17]. It works like the win ratio wherein patients, rather than events, are ranked using predefined, hierarchical criteria that account for both benefits and harms over a defined period. Although there are several advantages to a more holistic approach like DOOR, a key factor in implementing any of these methods is the credibility of the approach used to rank order various health outcomes.

## Approaches to deriving weights

To date, most applications of rank-based or weight-based methods have relied on consensus from small groups of clinicians [18], published utility weights used in cost-effectiveness analyses, or weights based on an event’s association with mortality [19]. However, several preference-elicitation methods can be used to estimate relative importance weights for endpoint events or health states, which also could be converted to rankings, from larger, more representative groups of stakeholders. One approach that can be easily implemented in a survey is a direct rating on a value scale. With this exercise, an arbitrary value, say 100 for death, is assigned to the most important event, and participants are asked assign lower or higher numbers to indicate relative importance. Another approach is a point-allocation, or constant-sum, exercise in which respondents are asked to allocate, say 100, points across a set of events based on their perception of their relative importance. A downside of this approach is that results also are dependent on the number and selection of events included. Electronic survey formats can help participants by showing the sum of points that have been allocated. Though these exercises are relatively easy to explain and administer, they require participants to complete tasks they rarely encounter in everyday life, and there is little theoretical basis to support either approach.

An alternative approach well suited to obtain relative importance weights for independent events or health states is object-case best-worst scaling (BWS) in which participants are shown several lists of, typically three to five, objects and asked to select the one that is most important (or severe, impactful, etc.) and the one that is least important (or severe, impactful, etc.) [20]. A central assumption with BWS is that people can more easily choose the extremes from short lists than assign a full ranking to longer lists of objects, particularly those that fall somewhere in the middle [21]. In this type of exercise, lists are predetermined using an experimental design to ensure that each object is shown and paired with other objects about the same number of times. Statistical models can be used to generate estimates of relative importance for all objects and associated 95% confidence intervals based on the number of times each is chosen as best or worst. A recently completed study demonstrated the use of BWS to estimate relative importance weights for nine clinical events commonly collected in clinical trials of anticoagulants from 1028 patients with atrial fibrillation. Findings revealed consistency in the rank order of importance of events across age, sex, and race groups, but relative importance weights varied by sex and race [20].

Discrete choice experiments (DCE) are widely used to quantify health preferences. In a DCE, experimentally designed hypothetical profiles are constructed using attributes selected to represent benefits, risks, and other relevant features. The profiles differ in regard to the levels shown for each attribute. For instance, an attribute representing the annual risk of a myocardial infarction could include levels of 0%, 1%, and 5%. In a DCE, respondents are shown a series of choice questions with each offering two or more constructed treatment or health-state profiles and asked to choose among the alternatives provided. Attribute levels such as magnitudes of risks or improvements in physical functioning are varied across profiles such that participants must forgo some desirable features as they evaluate choices. Attribute levels can be deterministic, representing definite outcomes such as mild bleeding or severe bleeding, or probabilistic, representing varying probabilities of outcomes. Due to cognitive limitations, DCEs are typically limited to about four to seven attributes [22], with a recommendation for smaller numbers of probabilistic attributes. This limits the number of different clinical events that could be studied using a DCE. Another concern about using DCEs to estimate relative importance weights is that the calculations rely on differences in preference weights across the range of attribute levels included in a study. As a result, an investigator could influence importance weights for attributes by selecting narrower or wider ranges of corresponding levels. One way to address this issue with probabilistic attributes is to calculate the relative importance of a one-percentage-point change in the risk of the event of interest relative to a one-percentage-point change in the risk of death [23].

**Figure 1.**
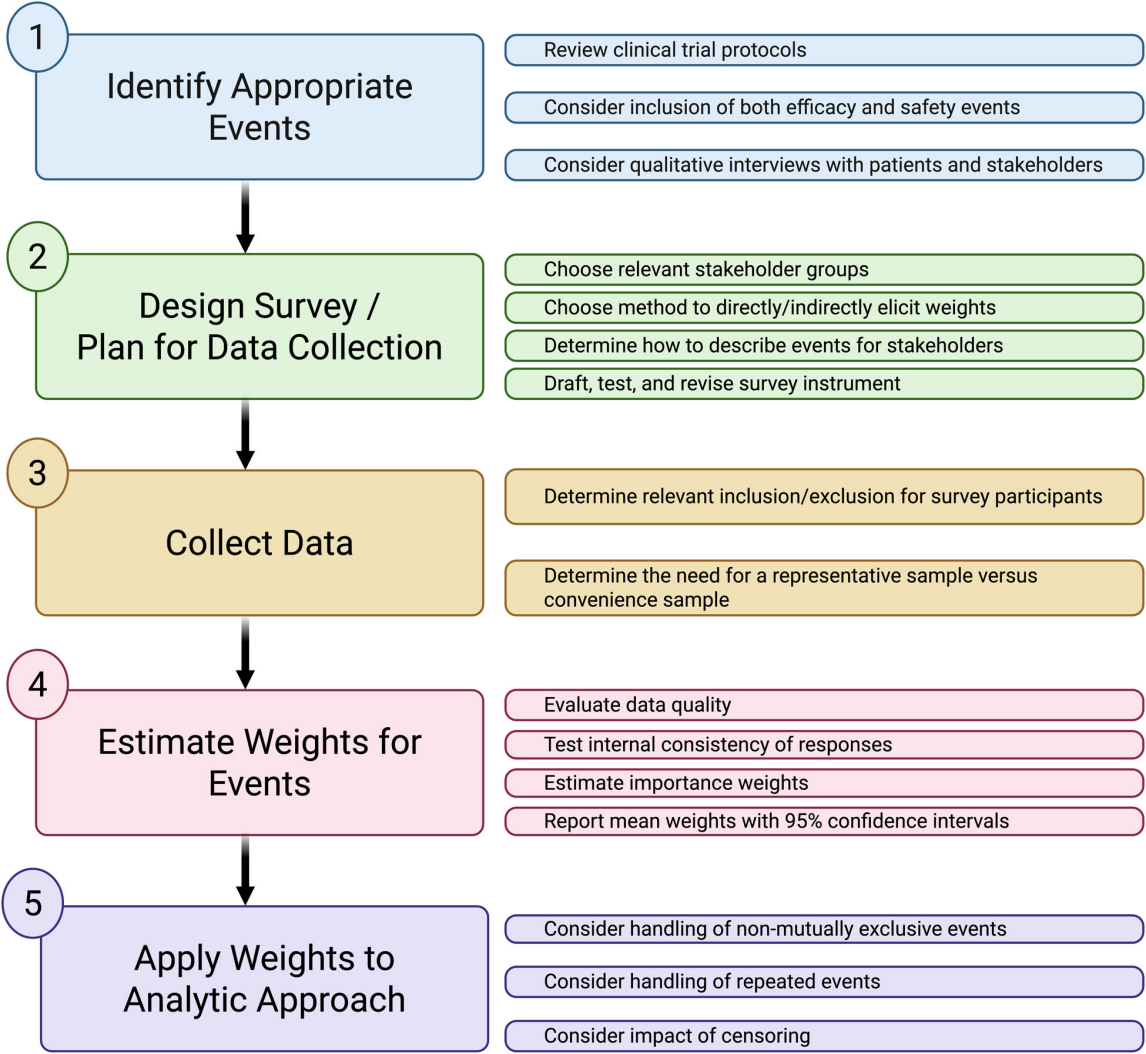
Steps in conducting analyses that account for relative importance of events.

Although the various approaches have strengths and weaknesses, survey-based methods provide transparent, objective, reproducible means to measure relative importance weights with estimates of uncertainty. Furthermore, surveys can be administered to various stakeholders to allow for comparisons of different sets of weights or rankings when applied to the same clinical data. This could elucidate areas where most physicians believe that the benefits of a treatment outweigh its harms and most patients believe otherwise, potentially forecasting where treatment uptake and adherence may be low. Regardless of the approach, several common issues must be carefully considered. Table 2 outlines several issues that should be considered when planning for an analysis that relies on weights, and the Figure 1 provides an overview of steps required to elicit and incorporate stakeholders’ views on the importance of events in analyses of clinical trial data.

**Table 2.** Options when estimating weights and applying them to clinical trial events

Selecting events for weighting	
Use clinical events that map directly to primary or secondary trial endpoints (e.g., stroke, death) Extend selection to include other events or outcomes that are meaningful to patients (e.g., adverse events, levels of physical functioning) Expand clinical events according to impact on resulting health status (e.g., stroke by modified Rankin Score)	
**Options for eliciting weights**	
Point allocation Object-case best-worst scaling Discrete choice experiment	
**Options for describing events**	
Describe the health event including the conditional probability of disabling outcomes or death (e.g., myocardial infarction with 5% risk of death within one year) Separately describe fatal and nonfatal events Describe as a defined clinical course for a patient (e.g., myocardial infarction with no residual impact on physical functioning at one year)	
**Options for scaling weights**	
Scale from 0 (least important event) to 1 (most important event) Scale relative to death (1) Scale such that the sum of weights equals 100 Scale events relative to the mean preference weight Scale events relative to 0 (dead) to 1 (perfect health) (e.g., utility weights)	
**Options for handling nonmutually exclusive events**	
Count only the first event Count only the most severe event Count a sequence of events as a single episode Count all events	
**Options for computing event occurrences**	
Compute percentages of patients experiencing one or more of each type of event Compute event rates that incorporate repeat events and observation time (# per patient-year), with the caveat that it will double-count repeat events in the same individual Compute incidence rates that incorporate the first event and observation time (% per patient-year)	
**Analytic approaches**	
Rank-based methods Win ratio Desirability of Outcome Ranking (DOOR)	Weight-based methods Multicriteria decision analysis Weighted win ratio Weighted nonparametric approach
**Options for handling potential differences in weights between clinicians and patients**	
Conduct separate analyses with group-specific weights and compare results Reach consensus on preference weights through deliberative processes like the Delphi method	

## Choosing events

There are several options for selecting “attributes” or endpoints to include in a preference-elicitation study. First, investigators could select attributes that directly map to medical events or health outcomes used as primary or secondary clinical trial endpoints such as myocardial infarction, stroke, and death, or expand the scope to include other outcomes that may be important to patients. This step initially seems straightforward. However, the relative importance of medical events extends beyond the immediate impact to include the associated mortality risk as well as mid- and long-term impacts on a patient’s mental and physical health status. For example, some patients experiencing a nonfatal myocardial infarction may undergo successful revascularization with little to no change in their health status, whereas other patients experiencing a myocardial infarction develop chronic heart failure and shortened survival. These two events differ in their relative importance to patients. Thus, it may be necessary to elicit separate weights for acute events that lead to differing sequelae. Investigators also must decide on how to handle deaths. One option is to describe the conditional probability of dying associated with each endpoint event (e.g., stroke with a 30% chance of dying within 30 days). This may be the most realistic but may be difficult for people with lower numeracy. Another option is to elicit weights for a nonfatal endpoint event and death separately. This approach assumedly is easier for patients and represents how endpoint events are frequently counted in clinical trials. Nevertheless, when clinicians are asked to weigh the relative importance of events, it is natural for them to inherently consider conditional risks of mortality and other sequelae associated with acute events. Thus, different ways of considering mortality risks could be an important factor in explaining why rankings/preferences differ between patients and clinicians.

## Importance of definitions

An important determinant of the credibility of survey-based preference-elicitation exercises is the extent to which respondents have a full understanding of the objects or attributes being evaluated. The relative importance of endpoint events, harms, and other factors is undoubtedly influenced by the descriptions provided in a survey. For patient surveys, it is necessary to avoid medical jargon and strike a balance between brevity and comprehensiveness. A review of descriptions for attributes representing stroke in patient preference studies reveals significant variability [23–25].

One problem that arises when testing descriptions of medical events occurs when patients make inferences about treatments that are required and health outcomes following the events based on personal experience. For instance, even if a survey includes a carefully crafted description of a disabling stroke, some people will infer that patients frequently experience a full recovery. This is reasonable given the variability in the extent to which people can be rehabilitated after a stroke. An approach that can be used to minimize the influence of assumptions people make is to describe the event about a specific patient concerning the treatments received, their clinical course, and their subsequent health. Then, in the survey, respondents are asked to consider the “people” who had specific events rather than the events [20].

Development and agreement on descriptions of frequently used endpoint events for use in patient surveys would facilitate comparisons across studies, but the field would also lose the capability of studying whether descriptive variations have systematic effects on patients’ valuations.

## Whose weights to weigh?

As previously highlighted, patients, providers, and health systems may value the relative importance of medical events differently. One could argue that because of healthcare providers’ knowledge about various medical events and their sequelae, their valuations of the relative importance of events may be more robust. However, this approach may be considered paternalistic and not informed by lived experience or the patient perspective. This discordance is evident in several surveys of physician and patient preferences. In the setting of coronary angiography, physicians ranked periprocedural death and long-term survival as the first and second most important outcomes. In contrast, patients ranked renal failure requiring dialysis as the most important outcome (ranked sixth by physicians), followed by periprocedural death as second and long-term survival as tenth [26]. It may be that physicians tend to underappreciate the impact of renal dialysis on quality of life and that some patients believe some fates are worse than death. Similarly, in the setting of therapeutic strategies for the management of dyslipidemia, patients prioritize the mode and frequency of intervention significantly higher than physicians who prioritize cholesterol reduction and side effects more than patients [27]. However, findings are not consistent across patient/physician groups and highlight the importance of lived experiences in making these value determinations [28–31]. Thus, the question remains, how do we reconcile the lived experience of patients with the medical expertise of physicians to create meaningful, patient-centric weighted composite endpoints? Clinical trials could utilize surveys to collect the preferences of enrolled patients and from clinician investigators that could be applied in separate analyses. Alternatively, clinical trials could take advantage of patient advisory boards to capture both clinician and patient perspectives, potentially using a Delphi approach to reach consensus.

## Analytic and reporting approaches for weighted composite endpoints

Once outcomes have been assigned a weight, they can be applied to trial data in various ways. One approach that is frequently used in quantitative benefit-risk analysis is multicriteria decision analysis (MCDA) in which relative importance weights are applied to event-specific incidence rates for benefits and risks in each treatment group to account for repeated events and variations in observation time across patients [20,32]. The weighted sum for each treatment group is compared and the group with the lower/higher sum is preferred (depending on whether more significant events are assigned higher or lower weights). Monte Carlo simulation can be applied to account for uncertainty associated with event rates and preference weights to calculate the percentage of times one treatment is superior to the other, an easily interpreted result. This approach works well when the events being considered are mutually exclusive and not part of a clinical sequence or disease progression; however, this is frequently not the case. In heart-failure trials, a potential composite outcome may include elevations in natriuretic peptide concentrations, heart-failure hospitalization, and cardiovascular death. The occurrence of these components is not mutually exclusive from each other, with elevated natriuretic peptide increasing the risk of heart-failure hospitalization and cardiovascular death. Several approaches can be used in the setting of nonmutually exclusive components of a composite endpoint. One approach is to include all events to potentially reflect a more global assessment of the risks and benefits [33]. An alternative approach is a time-to-first component event analysis, where predetermined weights are applied only to the first event [34]. Finally, a weighted analysis can be undertaken where the highest-weighted (most severe) event that occurred during the trial, or the highest-weighted event that occurred during a clinical episode, is analyzed. Numerous strategies highlighting the issues discussed above are available to help guide complex benefit-risk analyses with a patient-centric viewpoint, including the MCDA and the DOOR [17,35]. Table 3 summarizes the key characteristics of various approaches discussed throughout the paper.

**Table 3.** Key characteristics of analytic methods for analyzing multiple outcomes

	Conventional time-to-composite endpoint analysis	Win ratio	Weighted Win Ratio	DOOR^a^	Weighted nonparametric approach	MCDA^b^
Relies on rankings or relative weights for events	Neither	Rankings	Weights	Rankings	Weights	Weights
Accounts for multiple or repeated events for individual patients	No	No	Yes	Possible	Yes	Yes
Accounts for the timing of events	Yes	Yes	Yes	Possible	Yes	No
Occurrence of multiple lower-ranked (i.e., less important) events can compensate for a higher-ranked event	No	No	Yes	Possible	Yes	Yes
Can incorporate continuous outcomes, like biomarkers or QOL scores	No	Yes	Yes	Yes	Yes	Yes
Accounts for efficacy and safety events	No	No	No	Yes	No	Yes

a desirability of outcome ranking; ^b^multicriteria decision analysis. Disclaimer: Yes/no characterizations are based on typical use. Due to possible adaptations or use of more complicated scoring rules, yes/no characterizations will not always apply.

## Emerging analytic approaches

Various analytical approaches are emerging evaluate treatment effects that account for relative importance of events. One approach that lends itself well to time-to-event analysis is a weighted nonparametric approach [36,37]. In this type of analysis, each participant is assigned a score of 1.0 at time 0. In the event of a nonfatal event, the weight of that event is subtracted from their score (where larger weights represent more severe events). In the event of multiple, nonfatal events, the score is reduced multiplicatively (remaining score × weight of second event). Alternatively, weights can be multiplied with event-specific hazard ratios, resulting in a weighted all-cause hazard ratio [38]. In a composite scoring scheme, each nonfatal event is assigned a predetermined weight (e.g., heart-failure hospitalization – 0.2, recurrent myocardial infarction – 0.5) and death is assigned a weight that is equal to the maximal nonfatal participant score – 1.0 [39]. Each participant is then assigned a score that is equal to the sum of components of the composite endpoint, allowing comparisons between treatment arms. A comparison of these analytic approaches highlights a variety of strengths and weaknesses, including that none of these approaches account for the dependence of fatal events and recurrent events [40]. These approaches could potentially be improved with frailty models [41].

## Sample size calculations for weighted composite endpoints

Currently, there are very few clinical trials with weighted composite endpoints as the planned primary outcome, rather they are being used as part of secondary and exploratory analyses. As a result, there are not established approaches to direct trialists with regards to sample size calculations. At a minimum, these calculations would require consideration of event rates for each component, an assessment of the correlation between components and estimated effect sizes for all components of the composite endpoint. Previous studies have suggested that simulation models can be used to assist in these calculations [36], but further work is needed in this area.

## Conclusion

Adopting stated preference methods to systematically elicit the relative importance of treatment-related outcomes could help to advance the science of patient-centric endpoints. However, conscientious consideration of many aspects (Table 3) is required to ensure the results are clinically relevant, patient-centric, and statistically sound. Future studies should compare different elicitation and analytic approaches to better understand their impact on the interpretation of clinical trial results.
